# The effect of pre‐process and transport strategies on survival, microbiologic, and physiologic of *Patinopecten yessoensis*

**DOI:** 10.1002/fsn3.687

**Published:** 2018-06-14

**Authors:** Lanlan Pan, Chengxin Lin, GuoChen Zhang, Gang Mu, XiaoTong Yang

**Affiliations:** ^1^ College of Transport Equipment and Ocean Engineering Dalian Maritime University Dalian China; ^2^ College of Mechanical and Power Engineering Dalian Ocean University Dalian China

**Keywords:** depuration, live *Patinopecten yessoensis*, survival rate, temporary keeping, waterless‐low‐temp transportation

## Abstract

The supply chain of shellfish is complex, with animals being subjected to several stressors during the depuration, temporary keeping, and waterless‐low‐temp transportation processing. In this paper, the recycled water system for depuration and temporary keeping was used to realize both depuration and temporary keeping of *Patinopecten yessoensis*. The samples were divided into three groups based on three different pre‐process involved: samples in group 1 were depurated for 48 hr straight, whereas those in group 2 were first depured for 24 hr and then cooled for 24 hr; samples in group 3 was directly kept in a polyethylene insulation box. Then group 1 and group 2 were transported in a 3L polyethylene insulation box with ice packs (250 ml) to study the quality of transport based on the different pre‐process. As a result, in group 1 (depuration for 48 hr), the first death occurred after 56 hr, and all shellfishes died after 102 hr with total bacterial density of 2,630 CFU/ml. In group 2 (depuration for 24 hr and temporary keeping for 24 hr), the first death occurred after 104 hr and the total number of bacteria was increasing steadily within 0–104 hr. After 120 hr, all shellfishes died with total bacterial density of 1,090 CFU/ml. In group 3 (directly transport), all shellfishes died in 64 hr. The total number of bacteria in groups 1 and 2 declined significantly in the depuration process. The bacteria number (*p* < 0.05) in group 3 was significantly different from that in groups 1 and 2. The crude protein, crude fat, and glycogen of all groups declined. However, compared to groups 1 and 3, the consumption of glycogen in group 2 (*p* < 0.05) was delayed by the gradual cooling procedure. Those results prove that the depuration and temporary keeping procedures can improve the sterilization of the bacteria. The survival rate is less sensitive to the temperature change. The results provide satisfactory references for the *P. yessoensis*’ quality studies with depuration, temporary keeping, and waterless‐low‐temp transportation technologies.

## 1 INTRODUCTION

The scallop aquaculture industry has been steadily growing over the past decade, with over 2 million tons production in 2014 (FAO, 2014), The scallops are considered delicacies and healthy food items in several dietary regimes (Anacleto et al., 2014). Scallop can survive for extended periods out of water and those for human consumption are traded as live animals. The increased productions have improved the supply of this live product to markets worldwide, but distribution and sale of live mussel remains a complex issue (Barrento, Lupatsch, Keay, & Christophersen, 2013). During fishery transportation, the live shellfish should be stored in favorable living conditions for longer survival time. Typically, the survival rate declined over time affected by three steps, such as depuration, temporary keeping, and waterless‐low‐temp transportation (Anacleto, Maulvault, Barrento, et al., 2013; Anacleto, Maulvault, Chaguri, et al., 2013).

Shellfish depuration is a commercial compulsory process required in many countries for the fresh shellfish commercialization (Polo, Feal, Varela, Monteagudo, & Romalde, 2014). Several studies have been carried out to evaluate the effect of depuration in physiologic and microbiologic aspects of numerous shellfish species, such as *Crassostrea gigas* (Phuvasate, Chen, & Su, 2012; Phuvasate & Su, 2013), *Crassostrea virginica* (Larsen, Rikard, Walton, & Arias, 2015), *Ruditapes philippinarum* (Qiao, Cai, & Xu, et al, 2005), Meretrix Meretrix Linnaeus (Qiao, Cai, & Xu, 2008), *Patinopecten yessoensis* (Xian, Xin, Li, & Zhu, 2010), *Scapharca subcrenata* (Yang et al., 2012), *Mytilus edulis* (Barrento & Powell, 2016), which have provide the foundation for shellfish depuration process optimization of different temperature and water condition.

The amount and supporting facilities of depuration workshops still fail to satisfy the depuration requirements of shellfish for marketing (Polo et al., 2014). Shellfish cultured in decentralized aquaculture is often directly sold without being depurated. This is partially because the facilities and conditions of depuration are lacked, whereas the reason is more likely to be the concerns about potential increase in the death rate of the shellfish after the depuration. If the death rate indeed increases, losses will be caused. Yet, some research findings have proved that the death rate will not be increased by a proper environment and process for depuration. The survival rate of blue mussel (*M. edulis*) after depuration did not decrease before the transportation since the depuration of live mussel reduced the environmental stress (Barrento & Powell, 2016). The microorganisms and biochemical indexes of *Venerupis pullastra* (native clam) and *Ruditapes philippinarum* (exotic clam) during 2 days of depuration and subsequent transportation in semi‐dry conditions have been carried out, and the depuration has not affected the death rate, but reduced the total number of bacteria. The best semi‐dry transport conditions to maintain good physiologic conditions and high quality of clams is low temperature (4°C) (Anacleto, Maulvault, Barrento, et al., 2013; Anacleto, Maulvault, Chaguri, et al., 2013).

In the supply chain, scallop is subjected to several stressors during capture, depuration, storage, and transportation, including prolonged periods of air exposure and temperature fluctuation. The stressors strongly decrease the bivalves’ quality, cause some changes in the bivalves’ metabolism, and contribute to severe economic losses along the trade chain (Anacleto, Maulvault, Barrento, et al., 2013; Anacleto, Maulvault, Chaguri, et al., 2013).

Live fish have adopt gradient low temperature from high temperature to freezing‐point temperature to reduce the stress before waterless transport (Nie, Zhang, & Sun, 2014; Tian, Chen, & Yang, 2004; Zeng, Chen, & Shen, 2015). But no studies were performed with lower temperature step by step to connect waterless‐low‐temp transportation of scallop. The recycled water system was adopted in this paper to incorporate depuration and temporary keeping based on gradient cooling, to reduce the stress from high‐temperature depuration to low‐temperature transport of the scallop. Yesso scallop, *P. yessoensis*, are ubiquitous across eastern Asian coast; the main producer countries lie in North China and Japan with over 195,856 tons in 2014 (FAO, 2014). In this paper, the analytical results of quality characteristics were presented using the *P. yessoensis* samples to explore the efficiencies of the procedures from depuration, temporary keeping to waterless‐low‐temp transportation. Therefore, monitoring scallop quality is essential to avoid mortalities and optimize stake holders’ profitability, thus ensuring minimal quality losses to consumers and enterprise.

## 2 MATERIALS AND METHODS

### 2.1 Samples

Yesso scallops, *P. yessoensis*, with mean length of 85.3 ± 7.1 mm were purchased from Zhangzidao Group Ltd (Dalian, China), in December 2016 and were immediately transported to the laboratory in a cooling box without water. During a 120 hr simulated supply chain in laboratory, sampling was performed on the batch over numerous stages.

### 2.2 System and experimental setup

#### 2.2.1 Depuration and temporary keeping system

**Figure 1:**
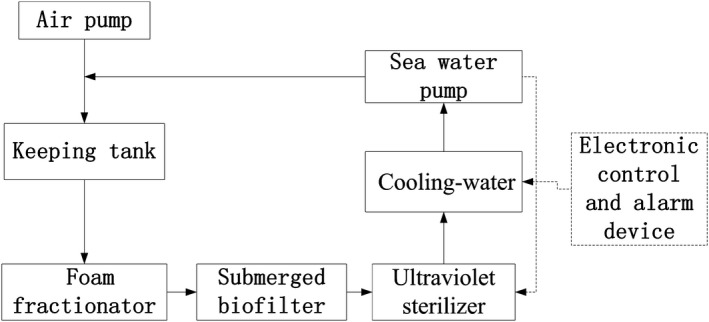
Process of recirculating aquaculture system for depuration and temporary keeping

The water‐recirculating system for depuration and temporary keeping includes three plastic tanks with each volume of 750 L which were utilized as the containers for depuration and temporary keeping scallops, submerged biofilter, foam fractionator, and UV sterilizer as purified water equipment. Water‐recirculating system was used sea water pump, air delivery system, and temperature control equipment for increasing oxygen and cooling water. The technological process of recirculating aquaculture system for purification and temporary keeping is shown in Figure 1. Waste water of the aquaculture flowing out of the temporary tank is purified and the temperature is controlled, Then, this water is injected into the temporary keeping tank to recycle water.

#### 2.2.2 Experimental setup

**Figure 2:**
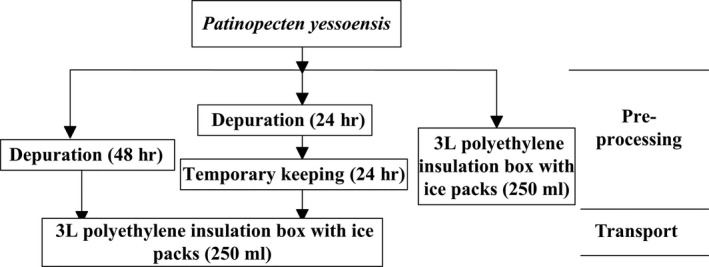
Schematic representation of experimental design

The sea water was stored after static segmentation in the depuration and temporary keeping system. The ultraviolet sterilizer (illuminance: 332 × 2 μ*W*/cm^2^) was adopted for disinfecting the sea water. The *P. yessoensis*, totally 300 animals were put into the depuration and temporary keeping after roughly selected, cleaned, and sorted. The water temperature of depuration was maintained at 15°C for 24 hr based on previous depurated condition researched (Croci, Suffredini, Cozzi, & Toti, 2002; Wang, Qin, Lin, Fei, & Zhang, 2008). In the temporary keeping, appropriate cooling rate and cooling time should be followed to avoid the stress reaction caused by the significant variation of temperature and by the reducing the survival rate. The temperature of water was cooled down at a rate of 3°C/h and then constant for 6 hr after reducing by 3°C in every state to realize gradient cooling. After this depuration and temporary keeping period, simulation of transport was carried out 3 L polyethylene insulation box with ice packs (250 ml). *P. yessoensis* (PY) undergoing different pre‐processing before transportation was split into three groups. The processing method of unitized pre‐processing and waterless transportation experiment is shown in Figure 2.

### 2.3 Physiologic analyses

#### 2.3.1 The survival rate

The survival recognition was performed by observing the closing of shell valves while stimulating the soft tissue with a stick (Paukstis, Janzen, & Tucker 1997). If the shell valve closed in 30 s, the individual was deemed alive.

$$\text{The}\;\;\text{survival}\;\;\text{rate}=\frac{\text{The}\;\;\text{survival}\;\;\text{number}}{\text{sample}\;\;\text{number}}$$

#### 2.3.2 Glycogen level analysis

Glycogen levels were analyzed using a commercial glycogen analysis kit (Shanghai Zhen Biotechnology Co, Ltd, Shanghai, China). Briefly, the soft tissue was overall homogenized and placed in a boiling bath with 1 ml alkaline solution for 20 min. An additional boiling bath for 5 min was performed after the chromogenic reagent of kit was added. Glycogen concentration was calculated by the absorbance at 620 nm of samples abiding by an equivalent glucose standard.

#### 2.3.3 Total viable counts

Microbial analysis is adopted to detect viable natural microorganisms in scallop meat. The aerobic plate count (abbreviated as APC) was detected, followed by the description of Gram (Gram, Trolle, & Huss, 1987). Three groups of 4 g samples were continuously diluted with sterile 0.85% NaCl solution, and 1.0 ml of each dilution was plated into duplicate plates of appropriate agar. Plate count agar (Beijing Land Bridging Technology Co. Ltd. Beijing, China) was applied for counting APC cells after incubation at 37°C for 48 hr.

#### 2.3.4 pH measurement

pH was measured using a pH meter (pHS‐3C Mettler‐Toledo, USA) with a spear tip combination electrode at 25°C. One gram of ground sample was diluted 1:10 (w:v) with distilled water and tested for pH (Cyprian et al., 2013).

#### 2.3.5 Crude protein and fat

The meat of two grams scallop was provided to measure the protein and fat, protein was ascertained using Kjeldahl nitrogen method (National Standard for P.R.C, 2016a,b), and fat was ascertained following Soxhlet extraction method (National Standard for P.R.C, 2016a,b).

### 2.4 Data collection and analysis

In transport state, a total of 100 scallops were average stock into 10 boxes of the each groups to avoid unpacking sampling in survival time, thus providing three repeated treatment every 8 hr for each trial group. SPSS 16.0 was adopted to statistically analyze the data. Data of each group were analyzed through one‐way analysis of variance and Duncan's method for multiple comparisons.

## 3 RESULTS AND DISCUSSION

### 3.1 Survival rate

**Figure 3:**
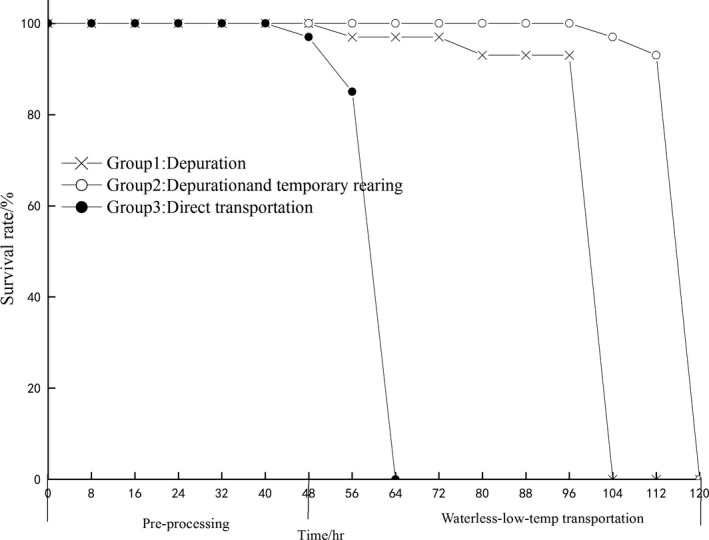
The survival rates of different pre‐processing and waterless transportation

Figure 3 shows the changes in survival rate of PY during the pre‐processing and transportation. During pre‐processing, no differences were found between groups 1 and 2 (survival rate always is 100%); however, a significant difference in survival of group 3 was observed, the death rarely occurred in 48 hr and the mortality of group 3 was 100% in 64 hr. After pre‐processing, samples in group 1 and 2 without water were raised in the polyethylene insulation box with ice packs, death rarely occurred in 56 hr and the mortality of group 3 was 100% in 104 hr. In group 3, death rarely occurred in 104 hr and the mortality was 100% in 120 hr. The survival time of group 2 was longer than that of group 1 by 16 hr.

During the depuration process, the number of bacteria or other pathogenic bacteria within the scallop was reduced, so the scallop thus became more adaptable to the environmental stress. Therefore, proper depuration and temporary keeping will not reduce the survival rate of shellfish during the transportation process (Anacleto, Maulvault, Barrento, et al., 2013; Anacleto, Maulvault, Chaguri, et al., 2013).

Group 1 from the high temperature environment with water suddenly entering into high temperature environment with exposed air has caused the death; group 2 has been treated by gradient cooling before entering into the waterless‐low‐temp transportation, so the temperature stress of PY is reduced and the survival rate is higher than that of group 1. Gradient cooling domestication can reduce temperature stresses of PY distributed in cold chain so that method has reduced the mortality and prolonged the survival time. However, but in this experiment, PY has low tolerance of exposed air stress, so their survival time is shorter than that of clam (Anacleto, Maulvault, Barrento, et al., 2013; Anacleto, Maulvault, Chaguri, et al., 2013), *Mytilus edulis* (Barrento, & Powell, 2016), and other bivalves (Phuvasate et al., 2012).

### 3.2 Total viable counts

**Figure 4:**
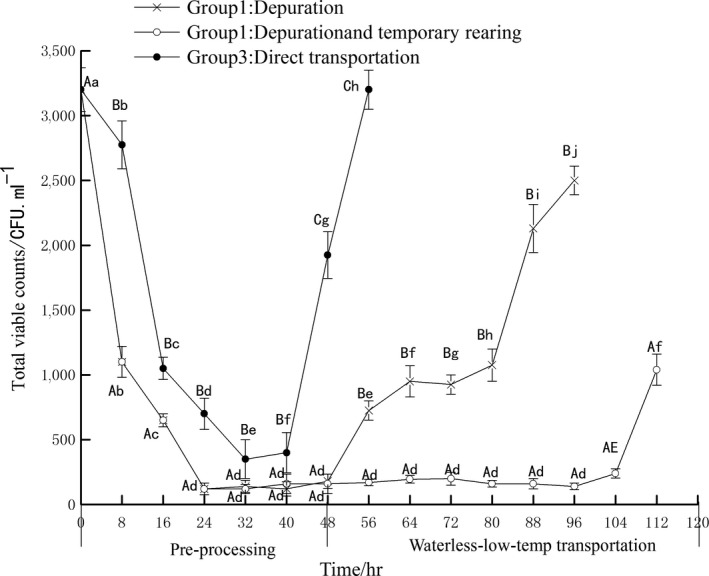
The change of bacteria number in different pre‐processing and waterless transportation

The total viable counts of three groups during pre‐processing and transportation were detected by APC. As presented in Figure 4, initial microbial counts took on a high microbiological load, as indicted 3,200 CFU/ml in control samples. Bacterial decrease accelerated with the increase in PY during 0–24 hr, but the bacterial counts for group 3 were significantly higher compared to groups 1 and 2.

Group 3's reduced number of bacteria was due to the low temperature environment, which was not favorable for the growth of thermophilic microorganisms and led to death of microorganisms. However, the decreasing speed of total bacteria was thus slower than PY in the depuration process. Therefore, the total number of bacteria in group 3 still kept on dropping even after the 24 hr until at 32 hr, but the bacterial change was not significantly different that at 32 and 40 hr (*p* > 0.05). However, bacteria grew significantly after 40 hr, and reached 2,970 cfu/ml at the 56 hr. The complex microbial community structure remained in PY without the depuration process, which causes an increase in the number of bacteria; so, some thermophilic microorganisms grew or died at fluctuated temperatures leading to the total bacterial growth.

During the depuration process of groups 1 and 2, total viable counts evidently decreased in 0–24 hr. The research complied with the study of *Ruditapes philippinarum* (Qiao, Cai, Xu, & Jiang, 2005) and *Meretrix linnaeus* (Qiao et al., 2008). UV disinfection has been more popular in recent years, ultraviolet disinfection can reduce the total bacteria in shellfish, and most common water disinfection methods adopted in commercial depuration are ultraviolet light (UV). Total viable counts of group 1 were still stable in 24–48 hr, so the 24–32 hr can be referenced as the shortest time for depuration of PY. Such results are consistent with the study by Croci and Wang (Croci et al.,^2002^; Wang et al., 2008).

Total viable counts of group 2 are not significantly different from that in group 1 in 24–48 hr (*p* > 0.05), and the temperature of temporary keeping process is lower than the optimum growth temperature of PY, so the low temperature reduced the body's metabolism and adverse impact to depuration (Chen, Ke, & Hong, 2012). However, the total viable counts of group 2 have been reduced to 120 cfu/ml after purification (0–24 hr), so the total viable counts did not change significantly (*p* > 0.05) at the 24–56 hr. The bacterial growth of group 1 accelerated over time during the transportation, particularly in 64 hr for the death of PY. At the 96 hr, the total bacteria reached 2,630 cfu/ml. On the contrary, the total number of bacteria in group 2 increased gradually. At the 112 hr, the total number of bacteria was only 1,090 cfu/ml. This was related to gradient cooling during the temporary keeping process, which not only reduced the environmental stress on live samples while entering the low‐temperature transportation process, but also maintained a low‐temperature environment. The total viable counts in *P. yessoensis,* after the depuration and temporary keeping process, grew slowly during the unitized transportation process.

### 3.3 Glycogen

**Figure 5:**
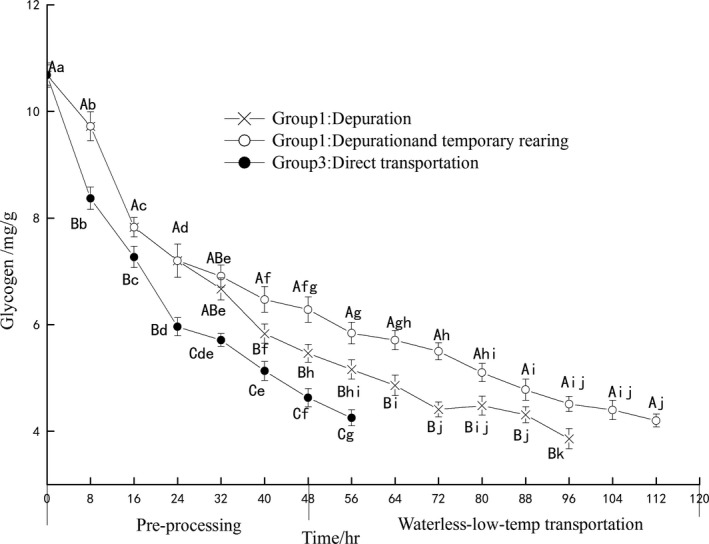
The change of glycogen content in different pre‐processing and waterless transportation

The glycogen levels of three groups during pre‐processing and transportation generally decreased (Figure 5). The overall effect of time to the glycogen levels of three groups shows statistical significance (*p* < 0.05). Glycogen is relatively critical for energy reserves (Du & Mai, 2004), and the glycogen level dropped in the face of stressors throughout the longest starvation (Riley, 1976). In this study, groups 1 and 2 always show significantly higher glycogen levels than the group 3 (*p* < 0.05). Long‐term environment of exposure air rapidly decreases the glycogen level of group 3, glycogen counts as the major carbohydrate reserve in mussel, and has been reported primarily used to maintain animal condition under stressful condition (Bayne, Thompson, & Widdows, 1976; Patrick, Faury, & Goulletquer, 2006). From 0 to 32 hr, the glycogen of group 1 and group 2 decreased along the time during the depuration, but group 2 has temporary keeping based on gradient cooling after depuration, from 32 to 112 hr, compared to group 1, the consumption of glycogen in group 2 (*p *<* *0.05) was delayed by the gradual cooling procedure.

### 3.4 Crude protein and fat

**Figure 6:**
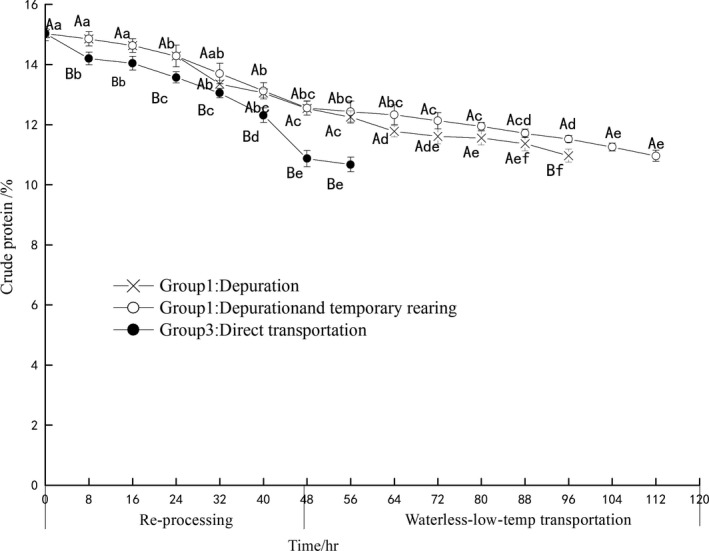
The change of crude protein in different pre‐processing and waterless transportation

The curve of crude protein levels of three groups is presented in Figure 6. The crude protein content under different pre‐processing methods is generally on a downward trend with passage of time. From 0 to 56 hr, the protein content of group 1 and group 2 differed significantly from that of group 3 (*p *<* *0.05). From 0 to 24 hr, group 1 and group 2 were in a water environment. Although live samples were not fed to maintain their hungry status, protein reduction in group 1 and group 2 was slower than group 3 directly entering the waterless environment. Many authors highlighted that applying the protein fraction as an energy source for maintaining bivalve metabolic should be merely realized when carbohydrate and lipid reserves have already been greatly depleted (Barber & Blake, 1981). The causes of the protein level variation are more complex than glycogen because protein is not just an energy‐reserving substance. Additionally, it does not serve as an energy‐reserving substance under some conditions. The altered protein levels in bivalves under certain stresses are variable depending on the species; for instance, *Mytilus edulis* (Dai, Zhang, & Zhang, 2014) and *Mactra veneriformis* (Jiang, Cui, Xia, & Bao, 2008) decreased protein levels at high temperature with the time, whereas oysters (*C. virginica*) indicated no alteration of protein level under the identical conditions (Ivanina, Dickinson, Matoo, et al 2013). Although PY showed low glycogen levels at the 112 hr. Thus, the authors argue that proteins may have been used to supply energy during waterless stress in PY.

**Figure 7:**
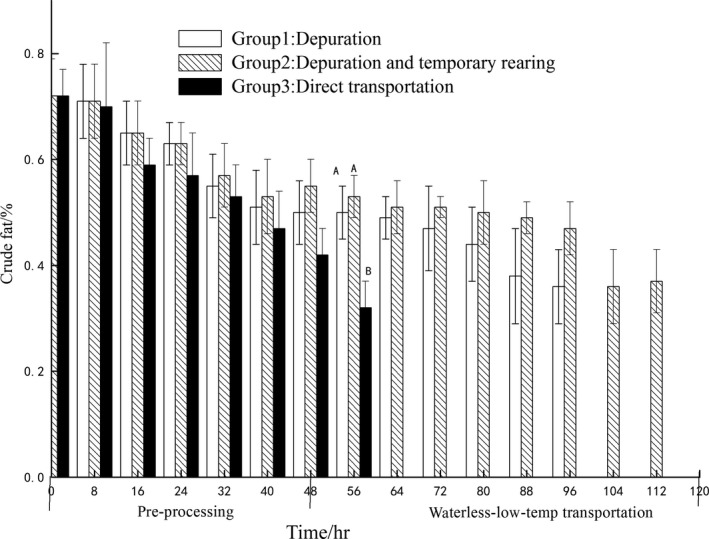
The change of crude fat in different pre‐processing and waterless transportation

The curve of crude fat levels of three groups is presented in Figure 7. Crude fat of three group samples showed no significant difference at 0–48 hr (*p* > 0.05), and at the 56 hr crude fat of group 3 was significantly lower than those of groups 1 and 2 (*p* < 0.05), the PY out water for a long period of starvation consequently decreased fat (Zheng et al., 2016). A significant difference in lipid content was observed in groups starved for 56 hr. The group 3 was suffering stress for keeping without water which could contribute to fat consumption. This could result in rapid fat mobilization in PY without water for a long time than compared with that in the water during starvation.

During the survival period, crude protein and crude fat of group 1 were not significantly different compared to group 2 (*p* > 0.05), PY stored in water of different temperature for a short time have not caused the crude protein and crude fat content changes.

### 3.5 pH value

**Figure 8:**
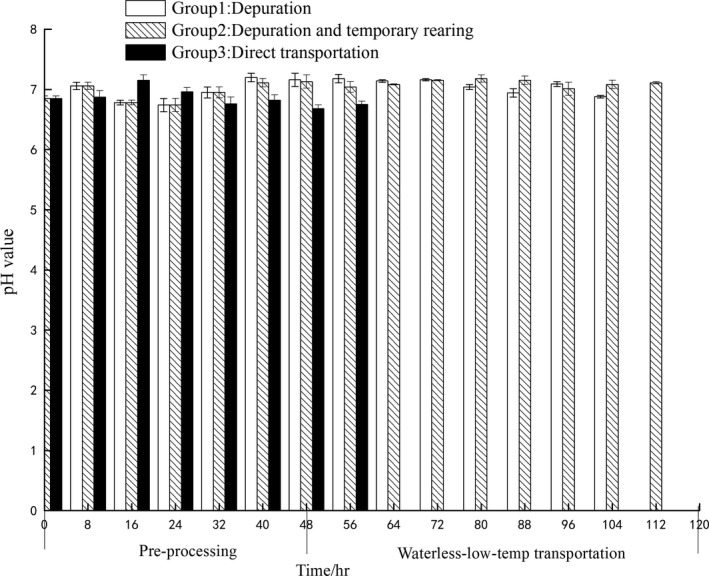
The change of pH value in different pre‐processing and waterless transportation

pH value is also a major index judging quality of aquatic products. The curve based on pH under different pre‐processing methods is shown in Figure 8. The initial pH value of live PY was around 6.85, which was close to neutral. During the depuration and temporary keeping, the pH value of group 1 and group 2 higher than their initial value because of the sea water. No statistical significance was observed in the pH values of the samples with different pre‐processing (*p* > 0.05), the changes of pH values were not observed significantly along time (*p* > 0.05). However, the pH value of all the three groups decreased accelerated nearby death. The decomposition of glycogen has produced lactic acid to bring down the pH value (Liu, Liu, & Ma, 2014); on the whole, the pH value of all the three groups ranged from 6.74 to 7.15.

## 4 CONCLUSION

During the fishery transportation process, the live shellfish should be raised in favorable living conditions for longer survival time. Typically, the survival rate declined over time, which is affected by water purification, temporary keeping, and waterless‐low‐temp transportation.

The result proved that the depuration and temporary keeping procedures can improve the sterilization of the bacteria. The survival rate is less sensitive to the temperature change. The survival time can be longer. With this experiment, the 24 hr depuration followed by 24 hr gradient cooling procedures is proved to be efficient for the waterless‐low‐temp transportation. The results provide satisfactory references for the *P. yessoensis*’ quality studies with purification, temporary keeping, and waterless‐low‐temp transportation technologies.

## ETHICAL STATEMENT

Animal welfare and experimental procedures were carried out in accordance with the Guide for the Care and Use of Laboratory Animals (Ministry of Science and Technology of China, 2006), and were approved by the animal ethics committee of Dalian Ocean University.

## CONFLICT OF INTEREST

None declared.
